# Atrial fibrillation and cognitive impairment: mechanisms, influencing factors, and prospects

**DOI:** 10.3389/fcvm.2025.1527802

**Published:** 2025-02-06

**Authors:** Li-Juan Huang, Jian-Shu Chen, Yu-Zhe Song, Peng Chang

**Affiliations:** ^1^ Second Clinical Medical College, Lanzhou University, Lanzhou, Gansu, China; ^2^ Department of Cardiology, Lanzhou University Second Hospital, Lanzhou, China

**Keywords:** atrial fibrillation, cognitive impairment, mechanisms, influencing factors, prospects

## Abstract

**Introduction:**

Atrial fibrillation (AF) is a prevalent cardiac arrhythmia and a significant contributor to cardioembolic stroke, a condition closely linked to cognitive decline. However, research reveals that AF itself is independently associated with an increased risk of cognitive impairment. This high incidence of cognitive decline in AF patients may result from various mechanisms, including reduced cerebral perfusion, microembolism, decreased cardiac output, and chronic inflammation.

**Methods:**

This review synthesizes current evidence on the relationship between AF and cognitive impairment, examines underlying mechanisms of cognitive decline in AF, and explores the roles of AF type, treatment approaches, left atrial characteristics, and associated conditions in cognitive function outcomes.

**Results:**

Cognitive outcomes in AF are further influenced by a range of factors, such as left atrial structural parameters, AF type and duration, anticoagulation use, catheter ablation, comorbidities, age, and gender.

**Discussion:**

The review highlights the complex interplay between AF and cognitive impairment, emphasizing the importance of understanding the various mechanisms and factors that contribute to cognitive decline in AF patients. By examining the roles of AF type, treatment approaches, left atrial characteristics, and associated conditions, this review provides insights into potential strategies for mitigating cognitive impairment in AF patients.

## 1 Atrial fibrillation and cognitive impairment

Studies show that cognitive impairment is notably high in AF patients, with an incidence of 8.70 per 1,000 person-years. Alzheimer's disease (AD) is the most prevalent form, comprising approximately 69.0% of cases, followed by vascular dementia at 21.8% and other dementia types at 9.0% (1).Atrial fibrillation is a well-established risk factor for post-stroke cognitive decline, with AF patients nearly three times more likely to develop dementia after a stroke than those without AF. This risk persists even when excluding a history of stroke (2).Preexisting cognitive impairment affects about 24.6% of AF patients prior to ischemic events (3).Patients with non-paroxysmal AF are at an elevated risk of cognitive impairment post-stroke (4). Persistent AF reduces brain activity in the frontal and temporal lobes, potentially impacting cognitive function (5).

AF is not only associated with vascular dementia but also linked to neurodegenerative diseases such as AD (1, 3, 6, 7). Furthermore, AF is considered a significant risk factor for Mild Cognitive Impairment (MCI). MCI is a transitional stage towards dementia, and early intervention is crucial for delaying or reversing cognitive decline (8). Cognitive function in AF patients, especially in terms of processing speed, declines more rapidly than in individuals without AF (9). The high incidence of cognitive impairment in AF not only impacts their daily lives but also imposes a considerable societal burden. Understanding the mechanisms and risk factors underlying cognitive decline in AF patients is essential for improving patient management and outcomes.

## 2 Mechanisms of cognitive impairment in atrial fibrillation

### 2.1 Neuropathological changes in brain tissue

Studies have shown that cognitive decline is highly prevalent among AF patients and is often linked to neuroimaging markers indicative of cerebral small vessel disease and neurodegenerative changes independent of ischemic stroke (Table 1). These markers include large infarcts, lacunar infarcts, silent brain infarcts, periventricular white matter hyperintensity (PVWMH), deep white matter hyperintensities (DWMH), and medial temporal lobe atrophy (MTA) (3, 10, 17). Such findings suggest a plausible connection between AF and cognitive impairment. Among AF patients with stroke, white matter hyperintensity (WMH) volume is significantly greater than in non-AF patients, suggesting an association between AF and increased cerebral burden (17).

**Table 1:** Summary of research on cognitive effects of brain tissue pathology in AF patients.

Author	Participants	Essential outcomes
Banerjee et al. (3)	Human	Pre-existing cognitive decline is independently associated with specific imaging markers on cranial MRI in patients with AF-related ischemic stroke or transient ischemic attack, including lacunar infarcts, increased periventricular WMHs, increased DWMH, and hippocampal atrophy.
Yoshihisa et al. (5)	Human	Patients with persistent AF show reduced brain activity in the frontal and temporal lobes, regions integral to cognitive function and closely linked with depressive symptoms. Those who underwent catheter ablation and successfully maintained sinus rhythm experienced an uptick in activity within these lobes, which coincided with reductions in depressive symptoms and improvements in cognitive performance. It indicates that persistent AF might influence cognitive and emotional well-being by altering activity in specific brain areas and restoring sinus rhythm through treatment may aid in enhancing those brain functions.
Nakase et al. (7)	Human	In comparison with patients in sinus rhythm, patients with AF have a larger volume of PVWMLs, and these lesions significantly impact the severity of cognitive decline. However, there is no significant difference in cerebral blood flow reduction. White matter lesions may be a more critical pathological mechanism leading to cognitive decline in patients with AF.
Rydén et al. (2)	Human	Patients with AF are more likely to exhibit cerebrovascular lesions, including symptomatic stroke, large infarcts, lacunes, and silent brain infarcts, potentially leading to cognitive decline. Moreover, these patients often present with a larger volume of WMHs, indicative of cerebral small vessel disease and further contributing to cognitive decline. Additionally, patients with AF have a higher prevalence of microbleeds in the frontal lobe, which correlates with both a decline in cognitive function and an increased risk of dementia.
Koh et al. (10)	Human	AF is linked to cerebral small vessel disease, including WMHs, cerebral microbleeds, and silent brain infarcts, potentially contributing to cognitive impairment. Patients with AF have a 38% higher risk of cerebral microbleeds, which in turn are associated with increased mortality and the risk of recurrent stroke. These associations support the theory that AF impairs brain health via cardiogenic embolism and cerebral hypoperfusion, underscoring the need for further research into strategies to prevent cognitive decline in this patient population.
Austin et al. (11)	Human	Intermittent AF is associated with reduced fractional anisotropy in white matter and an increased risk of microbleeds. Similarly, persistent AF is associated with a reduction in gray matter volume.
Wieczorek et al. (12)	Human	In patients with paroxysmal AF, cerebral WMHs are frequently observed, and their presence and severity correlate with age and the CHA2DS2-Vasc score. Cognitive function assessment using the MMSE revealed no significant differences in scores between patients with and without these hyperintensities.
Pommier et al. (13)	Human	Cognitive decline preceding AF-related ischemic stroke or transient ischemic attack is commonly observed, affecting about 24.6% of patients. This impairment is associated with neuroimaging markers of cerebral small vessel disease, including leukoaraiosis, cerebral microbleeds, and MTA, and predicts poorer functional outcomes at 24 months.
Salvadori et al. (14)	Human	In patients with AF, cognitive decline is associated with hyperintense white matter signals and MTA.
Tatewaki et al. (15)	Human	Regional cerebral blood flow significantly increased in the left posterior cingulate cortex after catheter ablation. Concurrently, there was a reduction in cortical thickness within the left posterior insular cortex. Cognitive assessments revealed improvements in domains such as executive function and verbal memory following treatment. These findings suggest that catheter ablation for AF may enhance cognitive function by modulating the autonomic nervous system. This leads to improved blood flow and structural integrity in brain regions implicated in cognitive function.
Johansen et al. (16)	Human	Atrial cardiomyopathy is significantly associated with the deposition of beta-amyloid protein in the brain, and this association remains after adjusting for demographic characteristics and risk factors. In contrast, AF is not associated with elevated levels of beta-amyloid protein in the brain.

Research also indicates that AF patients with cognitive impairment have larger periventricular white matter lesion (PVWML) volumes than those with sinus rhythm, with multivariate analyses linking AF to PVWMLs and more severe cognitive decline (7, 11). Additionally, intermittent AF (defined as AF episodes lasting less than 100% during ECG monitoring) is associated with reduced fractional anisotropy in white matter, suggesting microstructural changes related to cognitive function (11).

Findings from Pommier et al (13). indicate that AF patients present with greater volumes of white matter hypodensity, old cerebrovascular lesions, and cerebral atrophy on head CT scans, with cognitive impairment prior to stroke being independently associated with AF, even after adjusting for clinical and imaging variables. This association suggests that AF-related cognitive decline may not be solely attributed to visible brain damage. Salvadori et al (14). followed AF patients on anticoagulation therapy for 18 months and found cognitive decline in 27% of patients linked to WMHs and MTA, suggesting a role of MTA in AF-related cognitive impairment.

MTA may be a factor in AF -related cognitive impairment (3). Regarding temporal lobe involvement, the impact on cognitive function remains inconclusive. Some studies show that in patients with persistent AF who maintain sinus rhythm, temporal lobe activity improves three months post-catheter ablation, especially in those with preoperative deficits (5). Improved temporal lobe function correlates with higher Mini-Mental State Examination (MMSE) scores, although multivariate analysis has not demonstrated a strong independent association between AF and temporal lobe activity (5). Other studies found a significant decrease in left posterior insular cortical thickness six months after ablation, suggesting sinus rhythm restoration may protect cognitive function by affecting the central autonomic nervous system (15). Additionally, hippocampal atrophy, often associated with memory decline, is observed in some AF patients and linked to cognitive function decline (11).

**Figure 1:**
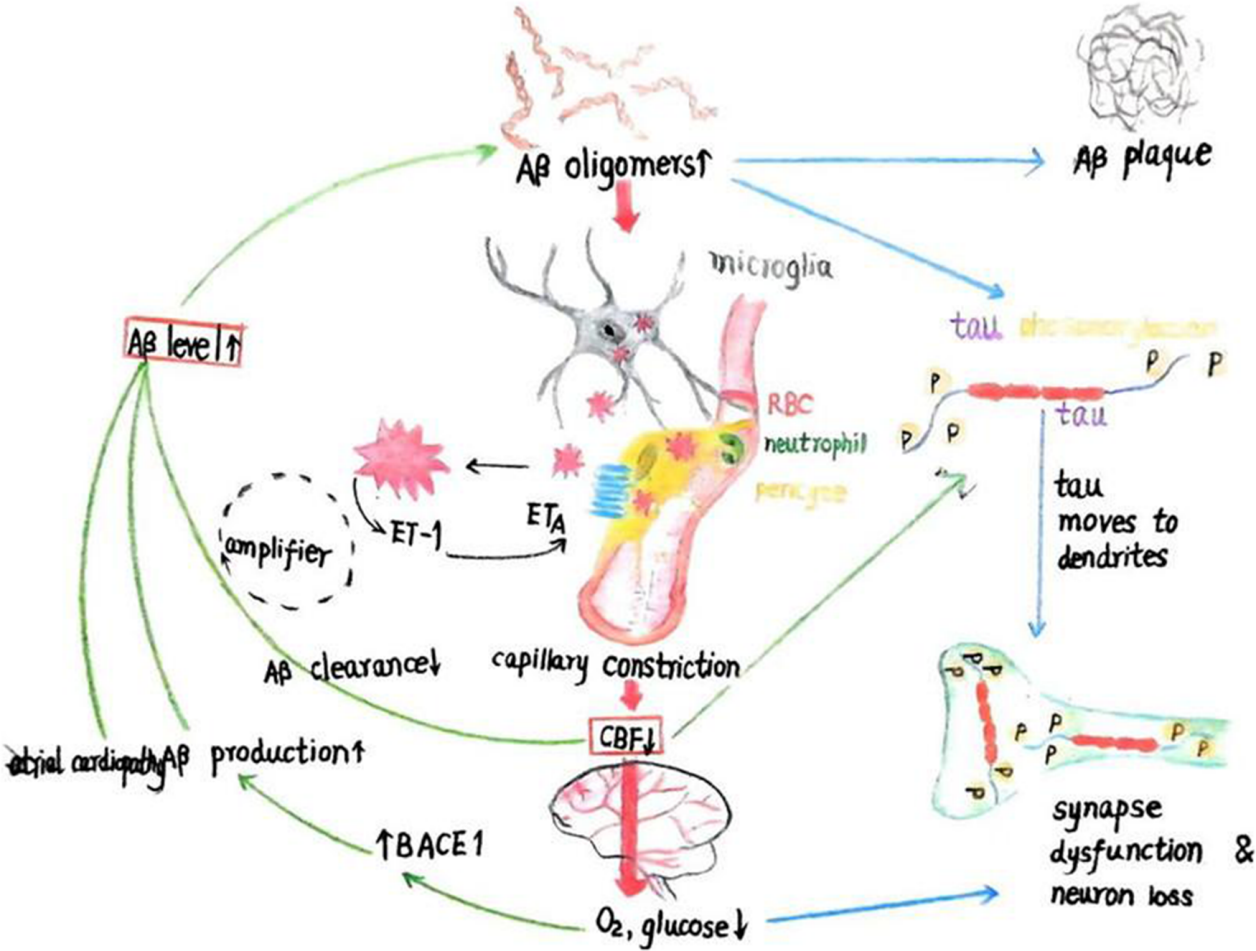
The schematic illustration depicts the initiation of amyloid-beta (Aβ) and tau cascades from two entry points (red boxes): (1) a reduction in cerebral blood flow (CBF), which leads to decreased cerebral O2 and glucose levels and upregulates the enzyme BACE1 that produces Aβ, or (2) increased Aβ levels due to enhanced production or diminished clearance. Aβ oligomers not only aggregate into plaques but also stimulate microglia and perivascular cells to produce reactive oxygen species (ROS), triggering the release of endothelin-1 (ET-1) from an unidentified cell type. The activation of ETA receptors on perivascular cells causes capillary constriction and a subsequent decrease in CBF, leading to reduced O2 and glucose levels. Elevated Aβ oligomer concentrations and diminished blood flow both contribute to the excessive phosphorylation of tau, which relocates from axonal microtubules to dendrites, causing synaptic dysfunction. This, coupled with myelin loss, results in cognitive decline. Additionally, the reduction in CBF is implicated in impaired cognition. (3) Atrial cardiomyopathy can lead to the deposition of beta-amyloid protein. Adapted from Korte et al. (57), licensed under CC BY 4.0

As AF may reflect underlying atrial cardiopathy, there is evidence that AF could be associated with beta-amyloid (Aβ) deposition, a hallmark of Alzheimer's disease (AD) (16). Atrial cardiopathy has shown a significant association with brain Aβ deposition, suggesting that AF may predispose some patients to AD-related pathology (Figure 1).

While substantial research links AF to pathological changes in brain structure and cognitive impairment, some studies provide contrary findings. Therefore, further research is needed to clarify the relationship between structural brain changes in AF and cognitive disorders.

### 2.2 Thromboembolism and stroke

AF is closely associated with cognitive impairment. Stroke is a known risk factor for cognitive decline. In post-stroke cohorts, patients with AF have a 2.70-fold increased risk of cognitive decline (10). Atrial fibril AF lation is also associated with silent cerebral infarction, suggesting that subclinical ischemic events may contribute to cognitive function decline (17, 18). Patients with both new-onset cerebral infarction and AF experience a greater decline in cognitive function than those with AF alone (19). Despite excluding individuals with a history of stroke at baseline and during follow-up, a significant association between AF and dementia persists (2). Patients with AF who experience a stroke are more likely to have had MCI or dementia beforehand (13). Studies have shown that the relationship between AF and dementia exists independently of stroke. Patients diagnosed with AF, whether during hospitalization or after discharge, exhibit a higher risk of dementia compared to those without AF (20). Weil et al. (21) found that an artificial intelligence-assisted ECG score >0.5 correlated with infarction and was also associated with poorer global cognitive function and a decline in attentional cognitive function at baseline. Additionally, cerebral microembolism was not linked to cognitive impairment; no significant decrease in MMSE scores was observed in patients with WMHs on magnetic resonance imaging (MRI) (12). Herber et al. (22) discovered that regular exercise correlates with a reduced incidence of ischemic stroke and WMHs, as well as an average increase of 0.08 standard deviation units in cognitive composite scores.

### 2.3 Reduced cardiac output and cerebral perfusion

AF may impair cognitive function via several mechanisms, including reduced cardiac output and diastolic cerebral blood flow (8, 10). Junejo et al. (23) first investigated the response of neurovascular coupling and the effects of AF on cerebral autoregulation in patients. They discovered that AF could elevate the risk of cognitive decline and stroke by disrupting neurovascular coupling and cerebral autoregulation. Consequently, these disruptions in cerebral blood flow regulation might significantly contribute to brain dysfunction in patients with AF. In a study by Kato et al. (24), patients who underwent catheter ablation for AF showed improvements in cardiac and cognitive functions six months postoperatively. This suggests that ablation may positively influence cognitive function by enhancing cardiac function and mitigating chronic cerebral hypoperfusion. These findings highlight the interplay between cardiovascular health and brain health. Tatewaki et al. (15) found that after ablation treatment for AF, the overall gray matter CBF ratio remained the same. Still, there was a significant local increase in CBF in specific brain regions, particularly the left posterior cingulate cortex. This increase correlated with cognitive improvements, particularly in executive functions and verbal memory. Patients with AF showed reductions in regional CBF in brain areas associated with AD pathology, suggesting that AF might contribute to cognitive decline by affecting cerebral blood flow. The study also found that reduced cardiac autonomic function in these patients was linked to cognitive decline, with a lower triangular index of heart rate variability independently associated with lower Montreal Cognitive Assessment (MoCA) scores, regardless of the cardiac rhythm at the time of assessment. Moreover, a higher average heart rate correlated with lower MoCA scores (25).

### 2.4 Inflammation and genetic factors

Cognitive dysfunction in patients with AF may be associated with inflammatory processes and genetic predispositions (18). Research has explored clinical variables and biomarkers associated with cognitive improvement following radiofrequency ablation, including plasma biomarker concentrations that are related to cognitive function and inflammation, such as adiponectin and leptin. The findings showed that, although baseline levels of these biomarkers correlated with cognitive impairment, they did not predict the improvement in MoCA scores one year post-ablation (26). The results do not conclusively link these factors to cognitive dysfunction. Insufficient evidence supports their role as inflammatory mechanisms in cognitive impairment associated with AF. Nakase et al. (7) conducted a study assessing white blood cell counts and C-reactive protein levels in patients with AF, but it failed to show their direct association with white matter lesions or establish a link to cognitive impairment. However, the study suggested that chronic inflammation might contribute to the development of white matter lesions, which are associated with cognitive decline. The APOE e4 allele is a known genetic factor linked to an increased risk of AD and plays a role in the metabolism and clearance of beta-amyloid protein in the brain. Some studies have found that the increased risk of dementia associated with AF is more pronounced in non-carriers of the APOE e4 allele, with no such association observed in individuals with at least one APOE e4 allele (2). Johansen et al. (16) mentioned the presence of the APOE e4 allele but found no significant interaction between this allele and the relationship between atrial cardiomyopathy and increased levels of beta-amyloid protein in the brain. This suggests that the effect of atrial myocardial disease on beta-amyloid levels may be independent of the APOE e4 genotype, though further research is needed to confirm this interaction. Studies indicate that interleukin-6 (IL-6) serves as an independent risk factor for the occurrence of atrial fibrillation (AF), playing a crucial role in both the initiation and persistence of AF. Particularly, individuals with the APOE e4 genotype may have an increased risk of Alzheimer's disease and greater severity of cognitive impairments, potentially due to enhanced IL-6 expression (27). Furthermore, the correlation between elevated fibrinogen levels and cognitive dysfunction is independent of the APOE genotype and the impact of prior cerebrovascular events. In women, higher levels of C-reactive protein (CRP) are significantly associated with reduced cognitive function (28). Carriers of the APOE e4 allele exhibit a marked increase in postprandial IL-6 levels after consuming a high-fat diet, suggesting a more robust postprandial inflammatory response in these individuals (29). The APOE e4 allele may influence cognitive function by affecting inflammatory responses, particularly through the modulation of tumor necrosis factor-alpha (TNF-α) levels (30). These findings elucidate potential biological mechanisms linking atrial fibrillation to cognitive impairment and offer new perspectives for future research and therapeutic approaches.

## 3 Mechanisms of cognitive impairment in AF

### 3.1 The influence of age and gender on cognitive function

Cognitive function in patients with AF is closely related to sex. Upon sex-stratified analysis, the association between AF and dementia was observed in males but not in females (2). However, the small sample size, particularly the limited number of female patients with AF, may have influenced the results. In another study, AF was significantly associated with symptomatic stroke, large infarcts, lacunar infarcts, and silent brain infarction in males, and there was a trend indicating a relationship between AF and large infarcts in females (17). A significant interaction between sex and AF affected delayed recall, indicating that females with AF experienced a greater decline in memory compared to those without AF (31). Female patients with AF were at a higher risk of developing dementia and MCI. They had an increased risk of progressing from normal cognition to MCI and from MCI to vascular dementia (32). Males exhibited a greater decline in expressive fluency, digit span, and performance on trail making test parts A and B over a two-year period compared to females (33).

### 3.2 The impact of left atrial parameters on cognitive function

Left atrial enlargement plays a pivotal role in the occurrence and progression of AF. Research indicates that cognitive impairment in patients with AF correlates with parameters related to the left atrium. After adjusting for covariates, including AF type, left atrial diameter is an independent predictor of cognitive impairment (4). Left atrial enlargement is also linked to MRI markers of cerebral small vessel disease in individuals without stroke or TIA history, including lower fractional anisotropy scores in white matter and the presence of microbleeds (11). Deposits of beta-amyloid protein, a hallmark of AD, are associated with an increased left atrial volume index (≥34 ml/m^2^), which predicts elevated levels of beta-amyloid protein in the brain and becomes more pronounced after adjusting for demographic characteristics and risk factors (16). Research also links a larger left atrial volume index to declines in Trail-Making Test A performance within a two-year period (34). Additionally, a lower left ventricular ejection fraction and reduced left atrial strain are associated with cognitive impairment (4, 11). Echocardiograms six months post-ablation reveal no significant changes in cardiac output or other parameters from baseline (15).

### 3.3 The impact of comorbidities on cognitive function

Studies show that comorbidities in patients with AF significantly affect cognitive function. Heart failure, diabetes, and a history of ischemic stroke are all associated with an increased risk of dementia in these patients (1). Univariate linear regression analysis reveals that factors significantly correlated with MoCA cognitive scores include age, diabetes, coronary artery disease, congestive heart failure, CHA2DS2-VASc scores, left atrial and left ventricular diameters, left ventricular ejection fraction, and the burden of AF (35). AF is strongly correlated with stroke, and studies consistently show a link between AF and cognitive decline in patients with a history of stroke, particularly affecting global cognitive function, learning, and memory (17, 31, 34, 36). After adjusting for various factors, including demographics and comorbidities, diabetes is associated with a small increase in the risk of dementia (34). Yet, in multivariate analysis, diabetes was not found to significantly affect cognitive decline (7). Research suggests that diabetes, AF, and the presence and severity of WMHs are treatable risk factors for cognitive impairment and dementia following stroke (37). Studies reveal a U-shaped association between systolic or diastolic blood pressure and dementia risk in middle-aged patients with AF, regardless of a history of stroke or oral anticoagulant(OAC) use; hypotension is associated with a higher risk of AD, while hypertension is associated with vascular dementia (1). Intensive blood pressure management increases dementia risk in patients with AF but reduces it in those without (38). This indicates that potential cognitive effects should be considered in AF treatment strategies. Research shows frailty or pre-frailty is often accompanied by cognitive impairment (36, 39, 40). Although AF is a risk factor for dementia, its presence does not significantly increase dementia risk in elderly individuals with existing ischemic heart disease (41). Lower hemoglobin levels are also independently associated with cognitive impairment, meaning that for each unit increase in hemoglobin, the likelihood of cognitive impairment decreases by approximately 17% (42). Asthma, arthritis, and cancer are associated with a reduced risk of dementia, which may be related to these patients’ more frequent contact with physicians, leading to earlier detection of cognitive impairment signs (34).

## 4 The impact of AF treatment on cognitive function

### 4.1 Catheter ablation

**Figure 2:**
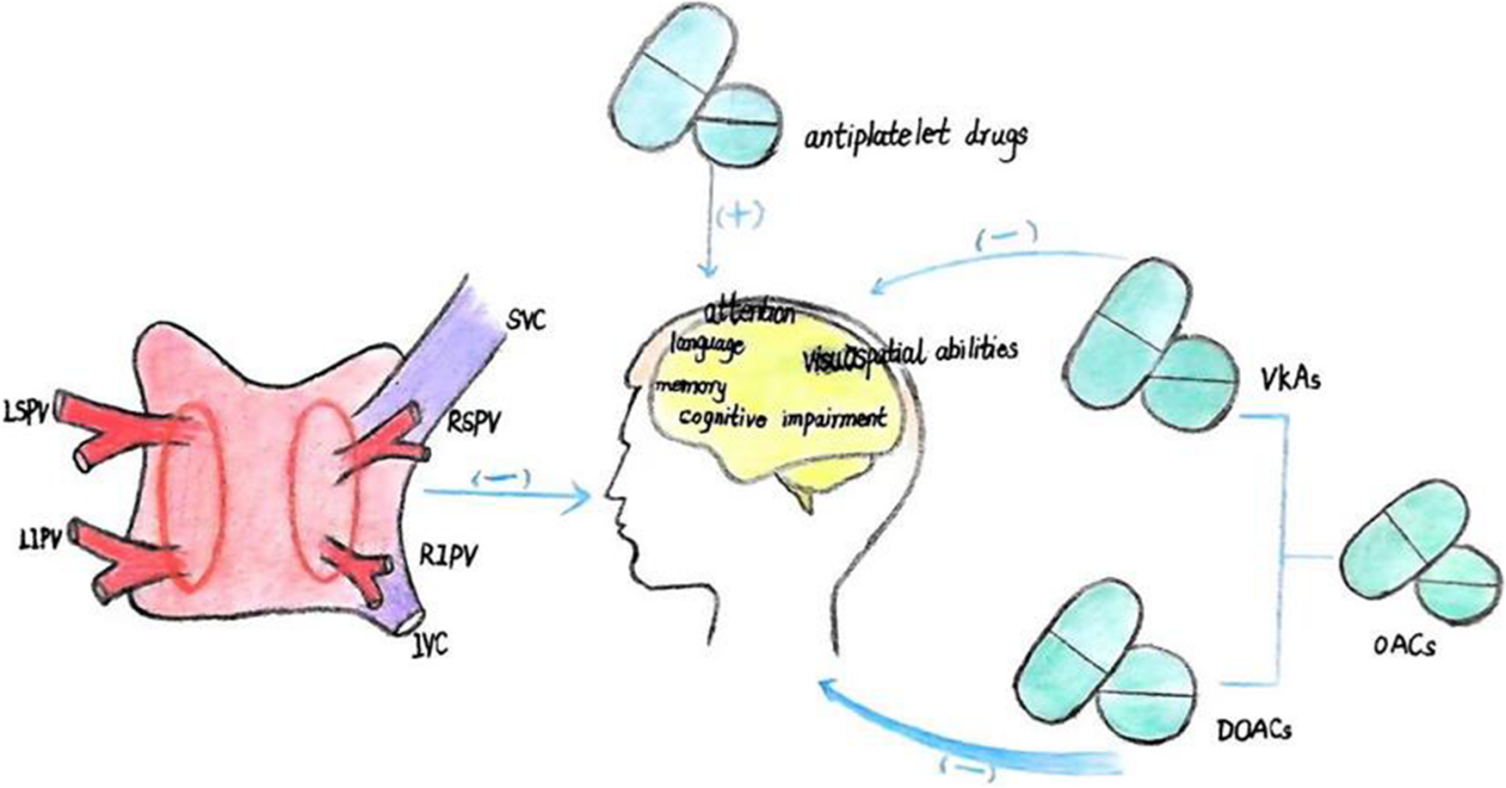
(1) catheter ablation enhances cognitive functions, notably in the areas of visuospatial ability, short-term memory, attention, and language. (2) OACs reduce the risk of cognitive dysfunction, with direct oral anticoagulants (DOACs) demonstrating superior efficacy compared to vitamin K antagonists (VKAs).In contrast, antiplatelet agents are associated with an increased risk of cognitive impairment.

Catheter ablation has been shown to positively impact cognitive function in AF patients (Table 2), with 12% and 14% of patients experiencing significant improvements at3 and 12 months post-procedure, respectively (45) (Figure 2). Long-term cognitive benefits have been observed in areas such as visuospatial abilities, short-term memory, attention, and language (46, 49). Studies suggest that these improvements may result from the maintenance of sinus rhythm following ablation. Notably, over half of the persistent AF patients who sustained sinus rhythm after catheter ablation demonstrated enhanced temporal brain activity three months after the procedure, especially among those with preexisting brain activity impairments (5).

**Table 2:** Summary of cognitive outcomes following catheter ablation for AF.

Author	Participants	Essential outcomes
Kato et al. (24)	Human	Studies suggest that, despite the potential for microembolism associated with ablation, the procedure has a protective effect on cognitive function. This is achieved by improving cardiac function, as evidenced by increases in ejection fraction, reductions in the left atrial volume index, and improvements in brain natriuretic peptide levels, as well as by alleviating chronic cerebral blood flow insufficiency.
Tatewaki et al. (15)	Human	Post-ablation, patients exhibit improvements in cognitive-psychological functions, particularly with significant increases in regional cerebral blood flow in the left posterior cingulate cortex and a reduction in cortical thickness in the left posterior insula. Research suggests that the left posterior cingulate cortex, as a key area of the limbic system, and the insula's crucial role in the central autonomic nervous system, these findings extend the hypothesis that changes in the autonomic nervous system are an important mechanism by which AF ablation positively impacts cognitive function. The study results indicate that AF ablation may improve cognitive function by affecting the autonomic nervous system.
Mohanty et al. (43)	Human	Overall, the study demonstrates the positive impact of catheter ablation on the cognitive function of patients with AF. The transient decline in cognitive function observed in a minority of cases shortly after surgery is likely a physiological response to the stress and tension of the procedure. By 12 months, no patients in the ablation group experienced a decline in cognitive function, compared to the pharmacological treatment group.
Rosman et al. (44)	Human	Patients with AF have shown significant improvements in cognitive function three months and one year after undergoing catheter ablation, particularly in visuospatial abilities, short-term memory, and language domains. Patients with impaired cognitive function at baseline exhibited the most notable improvements postoperatively. However, for those patients who experienced early recurrence of AF and failed to restore sinus rhythm, the improvement in cognitive function was not significant.
Wang et al. (45)	Human	For patients with AF, radiofrequency ablation and cryoballoon ablation therapies can improve cognitive function, primarily in specific domains such as memory, language, and attention, with benefits lasting at least 12 months. Moreover, both ablation methods are equally effective in enhancing cognitive function.
Guo et al. (46)	Human	Compared to rate control, rhythm control strategies significantly reduce the risk of future dementia, particularly AF ablation, which is associated with a significant reduction in the risk of overall dementia, AD, and vascular dementia.
Piccini et al. (47)	Human	Patients with AF who underwent catheter ablation and experienced no recurrence of arrhythmias showed more significant improvements in quality of life, cognitive function, and functional status compared to those with recurrences. Furthermore, regardless of whether there was a recurrence or not, cognitive function, as assessed by the MoCA score, improved postoperatively in all patients, suggesting a potential positive effect of ablation on cognitive function in patients with AF.
Takahashi et al. (48)	Human	Catheter ablation can significantly increase cerebral blood flow in patients with AF, particularly for those with non-paroxysmal AF. Moreover, having non-paroxysmal AF at baseline is an independent predictor of increased cerebral blood flow. Studies indicate that catheter ablation may have a positive effect on combating cognitive decline in patients with AF by improving cerebral perfusion, with particularly notable effects for those with non-paroxysmal AF.

Rhythm control therapy, as compared to rate control, has been associated with a significantly reduced risk of dementia in AF patients, with a hazard ratio of 0.74 (47) (Table 3). This suggests that rhythm control may lower the long-term risk of cognitive decline in AF (48). However, as observed by Rosman et al. (49), patients who experienced early AF recurrence after ablation and were unable to restore sinus rhythm with antiarrhythmic drugs did not show significant cognitive improvement. Interestingly, studies also indicate that catheter ablation alone can lead to cognitive improvements, as Piccini et al. found widespread cognitive benefits post-ablation, even in patients who experienced AF recurrence (50).

**Table 3:** Comparison of rhythm control and rate control strategies in alleviating cognitive decline.

	Advantages	Disadvantages
Heart rate control (43, 50)	Rate control strategies may be easier to implement. For patients who are not suitable for rhythm control surgery, rate control may be a safer option.	Rate control may not completely eliminate cognitive decline in patients with atrial fibrillation.
Rhythm control (43–46, 48–50)	Main outcome event risk reduction; improvement in cognitive scores, especially in the areas of visuospatial ability, short-term memory, and language; improved cerebral blood flow; especially for patients with non-paroxysmal atrial fibrillation.	Rhythm control may require more invasive treatments, which could increase the risks and costs associated with the therapy.

Compared to antiarrhythmic drug therapy, catheter ablation (including radiofrequency and cryoballoon ablation) offers superior cognitive outcomes for AF patients, particularly in memory function. Research indicates that radiofrequency and cryoballoon ablation are similarly effective in enhancing cognitive function, with no significant differences between the two methods (46). However, studies specifically examining the cognitive impacts of these surgical techniques remain limited.

Catheter ablation has demonstrated positive long-term effects on cognitive health. At six months post-ablation, patients showed increased MMSE scores and other neuropsychological improvements, reflecting gains across multiple cognitive domains (25). Additionally, catheter ablation led to greater increases in cerebral blood flow (CBF) compared to drug therapy, particularly in patients with non-paroxysmal AF at baseline, where the increase in CBF was most notable (51). After 12 months, the duration of the ablation procedure did not significantly influence cognitive outcomes, indicating that both ablation techniques provide comparable long-term cognitive benefits (46).

### 4.2 Anticoagulant therapy

Anticoagulant therapy can reduce the risk of cognitive dysfunction and may even improve cognition (Figure 2). Research has found that during an average 5.9-year follow-up period, the incidence of dementia/cognitive impairment in the OACs group and the non-OACs group was 12.1 and 13.3 cases per 1,000 person-years, respectively (50). A decline in cognitive ability was observed in 27% of elderly patients with AF, and this decline was associated with the duration of OACs intake (14). The use of OACs is associated with a 10% reduction in the risk of dementia/cognitive impairment (50). Some studies have found that in patients with a first ischemic stroke, AF diagnosed during hospitalization or after discharge, OACs can reduce the likelihood of developing dementia (20). Compared with treatment using VKAs; treatment with DOACs in patients newly diagnosed with AF is associated with a 16% reduction in the risk of newly diagnosed all-cause dementia (Table 4).

**Table 4:** The comparison of the impact of DOACs and VKAs on cognitive function.

	Advantages	Disadvantages
DOAC (12, 43, 44, 49)	Treating newly diagnosed atrial fibrillation patients is associated with a reduced risk of incident all-cause dementia; it is correlated with a reduced risk of new-onset mild cognitive impairment (MCI).	Bleeding risk, but lower than warfarin.
VKA (12, 43, 44)	There is no significant difference in the risk of cognitive impairment between warfarin and DOACs.	Warfarin may have a higher risk of major bleeding, requires regular INR monitoring, and dose adjustment is more complex. It is not as advantageous as DOACs in reducing cognitive impairment associated with atrial fibrillation.

Similarly, the use of DOACs is associated with a 26% reduction in the risk of new-onset MCI (42, 51). Compared with not using OACs, the use of OACs is associated with a 10% reduction in the risk of dementia or cognitive impairment. There is no significant difference in the risk of cognitive impairment between warfarin and DOACs; however, the use of both OACs and antiplatelet agents, compared with no treatment, is associated with a higher risk of dementia or cognitive impairment, possibly because patients receiving dual therapy have a higher risk of cardiovascular diseases, which may themselves be associated with a decline in cognitive function. Use of OACs, compared with no treatment, is associated with a lower risk of vascular dementia and dementia not otherwise specified (NOS), but not with the risk of AD (50). There is no statistically significant difference in the average change in cognitive function scores on the MMSE, NTB, and CGNT between the dabigatran and warfarin groups, although the MoCA cognitive score shows less cognitive decline in the warfarin group. However, this finding is not confirmed in the more comprehensive NTB and CGNT tests, so caution is advised when interpreting MoCA scores (52). Apixaban and edoxaban, compared with VKAs, are associated with a significant reduction in dementia risk; whereas dabigatran and rivaroxaban show no significant difference in dementia risk (53). Additionally, some studies have found that the use of antiplatelet agents instead of OACs is independently associated with cognitive impairment (42). However, other studies have reached different conclusions. A meta-analysis of 14 studies found that the relationship between AF and cognitive impairment could not be influenced by anticoagulant therapy, and when comparing anticoagulated AF patients to those who were untreated, there was no difference in the incidence of dementia (36). Another study indicated that the majority of AF patients were on anticoagulant therapy at baseline and continued throughout a 2-year follow-up period, yet new cerebral infarctions continued to occur frequently, suggesting that anticoagulant therapy alone may be insufficient to prevent brain damage and cognitive decline in all AF patients (19).

## 5 Summary and outlook

Current research in cognitive dysfunction is constrained by its reliance on singular assessment tools, such as the MoCA and MMSE, which may miss critical indicators and are susceptible to practice effects. Future studies must incorporate more robust cognitive assessment instruments and neuroimaging to improve early detection of cognitive impairment in AF patients. Moreover, research should encompass a broader demographic spectrum, including multicenter, multi-ethnic cohorts across various age groups and genders, to enhance the applicability of findings.

In anticoagulant therapy, while studies have evaluated the cognitive impact of different medications in AF, there is a paucity of research on how left atrial appendage occlusion affects cognitive function in these patients. The positive effects of catheter ablation are acknowledged, yet the distinct impacts of various ablation techniques warrant further investigation. Research has found that compared to isolated pulmonary vein isolation (PVI), PVI combined with additional ablation strategies, such as autonomic modulation (e.g., renal nerve denervation) and additional ablation lines, seems to enhance the effectiveness of treating atrial fibrillation.Gene editing technology and ablation techniques have complementary roles in treating atrial fibrillation and preventing cognitive decline. Gene editing technology may provide solutions at the etiological level, while ablation techniques play a role in symptom control and complication prevention (54). Current research predominantly focuses on treatment outcomes rather than the pathophysiology behind cognitive impairment in AF. Early identification of atrial fibrillation-related cognitive impairment is very important. Modal diagnostic models that integrate 3D MRI with amyloid PET imaging show high accuracy in the early diagnosis of AD, particularly in differentiating patients with a Clinical Dementia Rating of 0.5, achieving a 95% accuracy rate. Models that combine multimodal 3D imaging with graph convolutional networks also exhibit high accuracy and generalization capabilities in identifying MCI, which is essential for the early warning and intelligent diagnosis of AD. Future research will aim to enhance model performance, integrate 4D data to capture temporal dynamics, and identify the most diagnostically relevant time points (55, 56). Future endeavors should aim to elucidate the interplay between AF and cognitive impairment and develop more efficacious preventative and therapeutic approaches.
